# Correlation Between Changes in Extraocular Muscles and Intraocular Pressure Following Anti-Inflammatory Therapy in Active Thyroid Eye Disease

**DOI:** 10.3390/jcm14051480

**Published:** 2025-02-23

**Authors:** Yusuke Haruna, Mizuki Tagami, Mami Tomita, Atsushi Sakai, Norihiko Misawa, Kazuo Asano, Yusuke Murai, Atsuko Yoshikawa, Atsushi Azumi, Shigeru Honda

**Affiliations:** 1 Department of Ophthalmology and Visual Sciences, Graduate School of Medicine, Osaka Metropolitan University, Osaka-shi 5450051, Osaka-fu, Japan; yusukeh488@gmail.com (Y.H.); mami.dnc21@gmail.com (M.T.); mir.g.sail.arg.pneu.vet.asqm@gmail.com (A.S.); sannjaku@gmail.com (N.M.); shonda@omu.ac.jp (S.H.); 2 Department of Diagnostic and Interventional Radiology, Graduate School of Medicine, Osaka Metropolitan University, Osaka-shi 5450051, Osaka-fu, Japan; 3 Ophthalmology Department and Eye Center, Kobe Kaisei Hospital, Kobe-shi 6570068, Hyogo, Japan; y-murai@kobe-kaisei.org (Y.M.); monpey_696sp@yahoo.co.jp (A.Y.); azumi@kobe-kaisei.org (A.A.)

**Keywords:** thyroid eye disease, Graves’ ophthalmopathy, intraocular pressure, extraocular muscle, magnetic resonance imaging, steroid therapy

## Abstract

**Objectives**: We investigate the correlation between functional and morphological changes in extraocular muscles (EOMs) and intraocular pressure (IOP) changes before and after thyroid eye disease (TED) treatment. **Methods**: A multicenter study with a retrospective chart review was conducted. Patients with active TED receiving corticosteroid therapy without glaucoma eye drops between 2014 and 2023 were reviewed. Various parameters were measured by magnetic resonance imaging. The primary outcome measure was the correlation between changes in the IOP and the cross-sectional area (CSA) of the EOMs before and after treatment. Secondary outcome measures were comparisons of IOP, the signal intensity ratio (SIR) of the EOMs and orbital fatty tissue (OFT), and the CSA of the EOMs before and after treatment. **Results**: The IOP in 99 eyes in 51 patients significantly decreased from 18 ± 3.4 mmHg to 15.5 ± 2.9 mmHg before and after treatment (*p* < 0.01)). The CSA and SIR of all EOMs and OFT significantly decreased after treatment (*p* < 0.05). Factors that had a significant positive correlation with the IOP change rate before and after treatment were the CSA change rate of the inferior rectus muscle (IRM) before and after treatment (Spearman’s correlation coefficient, R^2^ = 0.24, *p* < 0.05) and the CSA change rate of the total EOMs before and after treatment (Spearman’s correlation coefficient, R^2^ = 0.22, *p* < 0.05). **Conclusions**: In TED patients, IOP decreased with anti-inflammatory treatment alone. The most significant parameter that correlated with the decrease in IOP was the CSA change rate of the IRM.

## 1. Introduction

Thyroid eye disease (TED) is an autoimmune disease of the orbital tissues that sometimes coexists abnormal thyroid function such as in Graves’ disease and Hashimoto’s disease [1,2,3]. Previous reports from the United States found that the overall prevalence of TED was 0.09%, and the prevalence was three times higher in women (0.12%) as compared to men (0.04%) at all ages [1]. Symptoms include double vision, orbital pain, eye movement disorders, eyelid swelling, upper eyelid retraction, exophthalmos, dry eyes, and hyperemia [2]. These symptoms are associated with the inflammation of the extraocular muscles (EOMs) and orbital fatty tissue, which are the results of an autoimmune process directed against the thyroid-stimulating hormone receptor [3]. In active TED, if thyroid dysfunction is present, it is important to restore thyroid function to normal using antithyroid drugs and then suppress the peak of inflammation with glucocorticoids, immunosuppressants, orbital radiation therapy, etc. [3,4,5].

Some TED patients also have ocular hypertension. Past studies have reported finding prevalence rates that were 8.5%, 24%, and 44% and which were higher than usual [6,7,8]. Previous reports have additionally shown that anti-inflammatory treatment with glucocorticoids lowers the intraocular pressure (IOP) in patients with TED that is accompanied by ocular hypertension [9]. This is thought to be related to an increased episcleral venous pressure (EVP) due to increased intraorbital volume [9].

At the present time, there has only been one report that has shown that there was a correlation between the width of the EOMs and IOP determined during an MRI examination [8,9]. Moreover, there have been no reports that have objectively proven that the IOP changes are due to changes in the intraorbital volume. In our present study, we evaluated IOP changes along with an evaluation of the inflammation associated with an anti-inflammatory treatment for TED by quantifying these changes, followed by an examination of the parameters in the orbit that were associated with the IOP changes.

## 2. Materials and Methods

Prior to this study, we obtained approval from the Ethics Committees of the Osaka Metropolitan University (OMU) (approval no. 2023-072) and the Kobe Kaisei Hospital (KKH), Japan. The study protocol was a multicenter study with a retrospective chart review of all patients who met the study criteria described below. All patients at OMU who participated in the study provided optout informed consent for their patient information to be stored in the hospital database and used in this study, and we have obtained optout consent from all patients at KKH. This study was conducted in accordance with the tenets of the Declaration of Helsinki. Treatment protocols were as follows for each facility.

### 2.1. OMU

Patients were treated with a cumulative dose of 6 g of methylprednisolone divided into 3 weekly doses, combined with orbital radiotherapy of 20 Gy in 10 divided doses. In other words, one course (one week) consisted of administering 1 g/day for 3 days, followed by a 4-day washout period, and a total of 2 courses (2 weeks) of administration were performed. Subsequently, they then started taking 30 mg of methylprednisolone, with tapering off performed at a pace of 5 mg/month over 6 months.

### 2.2. KKH

Patients were treated with a cumulative dose of 9 g of methylprednisolone divided into 3 weekly doses, combined with orbital radiotherapy of 20 Gy in 10 divided doses. In other words, one course (one week) consisted of administering 1 g/day for 3 days, followed by a 4-day washout period, and a total of 3 courses (3 weeks) of administration were performed. Subsequently, they then started taking 30 mg of methylprednisolone with tapering off performed at a pace of 5 mg/month over 6 months.

From October 2020 to May 2023 at the Department of Ophthalmology, OMU, and from July 2014 to April 2017 at the Department of Ophthalmology, KKH, patients who completed the corticosteroid therapy (combined with orbital radiotherapy) for TED and who met the following protocol were selected. Treatment was started regardless of thyroid function depending on the degree of TED.

The inclusion criteria were as follows: (1) patients who underwent the above treatment protocol for moderate-to-severe TED and (2) patients who underwent orbital MRI examination (coronal section, short-inversion-time inversion recovery (STIR) method) before and after the treatment protocol. The exclusion criteria were as follows: (1) patients who were receiving glaucoma eye drops before and after treatment; (2) patients who underwent ophthalmic surgery during treatment, such as in the form of strabismus surgery or orbital decompression surgery; (3) patients who discontinued treatment according to the following protocol for any reason; and (4) patients with irregular astigmatism.

TED was diagnosed at each hospital. All patients had Basedow’s disease and received medication by a physician. The diagnosed severity was based on the European Thyroid Association/European Group on Graves’ Orbitopathy (EUGOGO) management guidelines [10], with the corticosteroid therapy (combined with orbital radiotherapy) performed for moderate-to-severe TED. All patients treated had a clinical activity score (CAS) of 3 or above.

**Figure 1:**
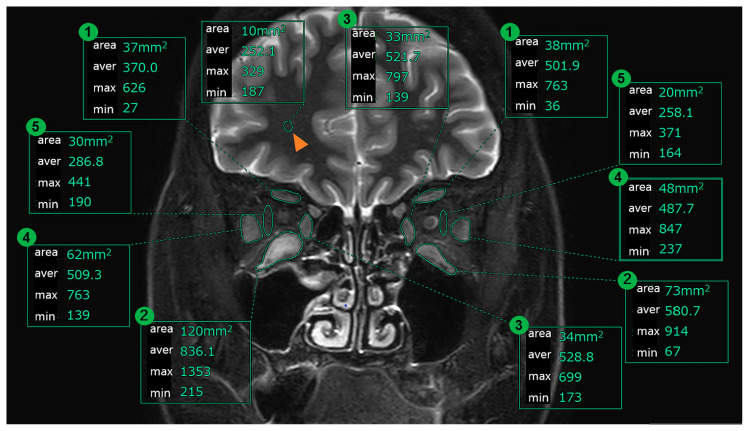
Coronal MRI and measurement method. Note—area: cross-sectional area (mm^2^), aver: average of STIR signal intensity, max: maximum of the STIR signal intensity within the selection, min: minimum of the STIR signal intensity within the selection, orange arrows: white matter, 1: SRM, 2: IRM, 3: MRM, 4: LRM, and 5: orbit fatty tissue.

We measured the IOP, cross-sectional area, and signal intensity ratio (SIR: as explained below) of the EOMs before and after the treatment protocol, then examined the changes before and after treatment and the correlations that were observed between them. We selected both eyes in each patient. The IOP was measured using a non-contact tonometer (Nidek Co., Ltd., Aichi, Japan) in the primary position of gaze in all patients. Pre-treatment IOP measurements were performed at a visit within 1 month before and after the MRI scan. Post-treatment IOP measurements were performed at a visit within 1 month after the day of MRI scan. Orbital plain MRI examinations were performed within the several months before the treatment and within the 1-month period after the end of the treatment protocol. Coronal sections were imaged in 3 mm slices using imaging conditions that included the STIR method. The STIR method is an imaging method that suppresses the signal from the area where the T1 is shortened by making the T1 shorter than the normal IR method and additionally emphasizes both the T1 and T2 in other areas [11,12]. Higher contrasts can be obtained in areas with a lot of fat such as inside of the orbit. We selected coronal cross-sections of the images that were taken in 3 mm slices using the STIR method. Among these, we then selected coronal cross-sectional images that were taken approximately 9 mm behind the edge of the eyeball (3 slices from behind the last cross-section in which the eyeball appears). Three ophthalmologists and one radiologist (Y.H, A.S, N.M, and K.A.) evaluated and obtained the images from the areas that surrounded the EOMs (superior rectus: SRM, inferior rectus: IRM, lateral rectus: LRM, and medial rectus: MRM) and then measured the internal cross-sectional area and signal intensity. We also evaluated images from certain areas that surrounded the orbital fatty tissue between the LRM and the eyeball, then measured the signal intensity inside the area (Figure 1). We used dedicated analysis tools (Synapse Vincent, Fujifilm, Minato-ku, Tokyo, Japan). In addition, we measured the signal intensity of the white matter within a 10 mm range in the same cross-section, with definition of the SIR as presented below.

Signal intensity ratio (SIR) = EOM signal intensity or orbital fatty tissue signal intensity/white matter signal intensity.

In addition, we defined the sum of the CSAs of the four rectus muscles as the CSA of the TRM (total rectus muscles). We defined the average of the SIRs of the four rectus muscles as the SIR of the TRM. The diameter of the superior ophthalmic vein (SOV) was also measured. The diameter of the section with the greatest dilation was manually measured on the coronal cross-sectional images of the orbital MRI (STIR method). The degree of proptosis was measured using the T1 axial image of MRI and was defined as the perpendicular distance between the line connecting the outer edges of both orbits and the corneal apex.

The primary outcome measures were (1) correlation between the IOP change rate (IOP before treatment-IOP after treatment/IOP before treatment) and the CSA change rate in the cross-sectional area of the EOMs (CSA before treatment-CSA after treatment/CSA before treatment) and (2) correlation between the IOP change rate and the SIR change rate of the EOMs, and correlation between the IOP change rate and the SIR change rate of orbital fatty tissue (SIR before treatment-SIR after treatment/SIR before treatment).

Secondary outcome measures were (1) IOP before and after treatment, (2) SIRs of the EOMs and orbital fatty tissue before and after treatment, and (3) CSA of the EOMs before and after treatment.

### 2.3. Statistical Analysis

All statistical analyses were performed with EZR [13] (Version 1.60, Saitama Medical Center, Jichi Medical University, Saitama, Japan), which is used for the calculation of R. More precisely, it is a modified version of R commander, which is designed to add statistical functions frequently used in biostatistics. Student’s paired *t*-test was used to test for significant differences in the mean values between the two groups, with the correlation between the measured values of the various quantities examined using Spearman’s correlation coefficient. Values of *p* < 0.05 were considered statistically significant. The Kruskal–Wallis test was used for comparison between three groups.

## 3. Results

In total, 104 eyes of 52 TED patients were included. At OMU, 54 eyes from 27 TED patients were included, while at KKH, 50 eyes from 25 TED patients were included. One eye in one patient was excluded as the patient had a prosthetic eye. Two eyes were excluded due to the fact the patients were using glaucoma eye drops. And two eyes in one patient were excluded due to irregular astigmatism. All patients selected for inclusion are summarized in Table 1. This study evaluated 99 of 51 TED patients with 11 male patients and 40 female patients included in the evaluation group. The mean age was 54.5 ± 12.9 years, and the mean IOP was 18 ± 3.4 mmHg. The mean spherical equivalent (SE) was −2.4 ± 2.8. Hyperopia (≤0D, including emmetropia) was 23%, mild myopia (>−3.0D, <0D) was 39%, moderate myopia (>−6.0D, ≤−3.0D) was 27%, and severe myopia (≤−6.0D) was 10%. There were 27 patients in the 6 g of methylprednisolone treatment group (6 g group). The mean age was 53.8 ± 12.5, the mean IOP was 17.9 ± 3.7 mmHg, and the mean SE was −2.2 ± 2.9 D in the 6 g group. There were 24 patients in the 9 g of methylprednisolone treatment group (9 g group). The mean age was 54.3 ± 12.8, the mean IOP was 18.2 ± 2.9 mmHg, and the mean SE was −2.6 ± 2.7 D in the 9 g group. There were no significant differences in sex, age, IOP and SE between the 6 g group and 9 g group. In addition, the correlation coefficients for the SE and various parameters were evaluated. There was no significant correlation with the SE for the IOP change rate, the CSA change rates of all EOMs (SRM, LRM, LRM, MRM, and TRM), and the SIR change rates of all EOMs (SRM, LRM, LRM, MRM, TRM, and OFT) (Spearman’s correlation coefficient, *p* > 0.05). A total of 95 out of 99 eyes were measuring FT4 within one month of treatment with an average of 1.3 ± 0.8 ng/dL (maximum: 5.26 ng/dL, minimum: 0.47 ng/dL). Hyperthyroidism was observed in 18 eyes (18.9%) and the average of FT4 was 2.7 ± 1.1 ng/dL. Only two eyes exceeded 5 ng/dL. Euthyroid was observed in 63 eyes (66.3%) and the average of FT4 was 1.2 ± 0.3 ng/dL. Hypothyroidism was observed in 14 eyes (14.7%) and the average of FT4 was 0.7 ± 0.2 ng/dL. There was no significant difference in the SIR between all EOMs, CSAs of all EOMs, and IOP before and after treatment among the three groups: hyperthyroidism, euthyroidism, and hypothyroidism (*p* > 0.05).

**Figure 2:**
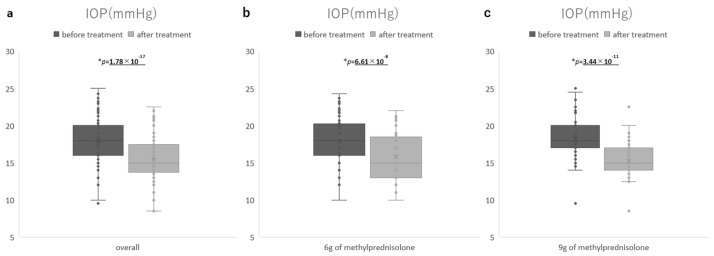
IOP before and aftertreatment: (**a**) overall, (**b**) 6 g of methylprednisolone group, and (**c**) 9 g of methylprednisolone group.

The IOP significantly decreased from 18 ± 3.4 mmHg to 15.5 ± 2.9 mmHg after treatment (paired *t*-test *p* = 1.78 × 10^−17^) (Figure 2). In the 6 g group, the IOP significantly decreased from 17.9 ± 3.7 mmHg to 15.7 ± 3.3 mmHg after treatment (paired *t*-test *p* = 6.61 × 10^−8^). In the 9 g group, the IOP significantly decreased from 18.2 ± 2.9 mmHg to 15.3 ± 2.6 mmHg after treatment (paired *t*-test *p* = 3.44 × 10^−11^) (Figure 2).

Moreover, when the IOP changes before and after treatment were compared between the normal IOP group (having a before-treatment IOP lower than 20 mmHg) and the high IOP group (having a before-treatment IOP greater than 20 mmHg), both groups achieved a significant decrease in the IOP due to the treatment (*t*-test *p* = 0.02 × 10^−11^, *p* = 0.02 × 10^−4^).

**Figure 3:**
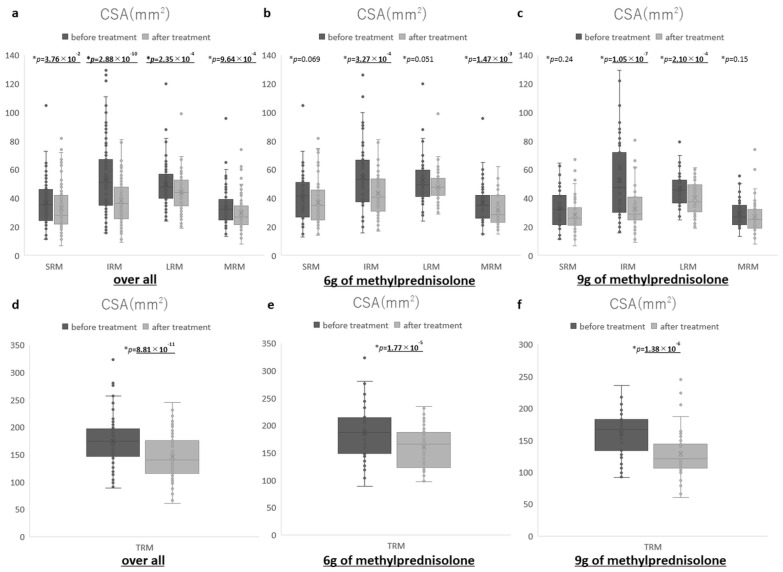
Comparison of CSA parameter before and after treatment: (**a**) overall (SRM, IRM, LRM, MRM), (**b**) 6 g of methylprednisolone group (SRM, IRM, LRM, MRM), (**c**) 9 g of methylprednisolone group (SRM, IRM, LRM, MRM), (**d**) overall (TRM: sum total), (**e**) 6 g of methylprednisolone group (TRM: sum total), and (**f**) 9 g of methylprednisolone group (TRM: sum total). TRM: total rectus muscle. OFT: orbital fat tissue.

Before and after the treatment, the CSAs of the EOMs significantly decreased from 36.8 mm^2^ to 33.1 mm^2^ in the SRM, from 53.7 to 38.2 mm^2^ in the IRM, from 49.9 mm^2^ to 44.2 mm^2^ in the LRM, from 33.3 mm^2^ to 29.7 mm^2^ in the MRM, and from 174.1 mm^2^ to 145.2 mm^2^ in the TRM (paired *t*-test SRM: *p* = 3.76 × 10^−2^, IRM: *p* = 2.88 × 10^−10^, LRM: *p* = 2.35 × 10^−4^, MRM: *p* = 9.64 × 10^−4^, TRMA: *p* = 8.81 × 10^−11^). In the 6 g group, before and after the treatment, the CSAs of the EOMs significantly decreased from 55.0 to 43.2 mm^2^ in the IRM, from 36.8 mm^2^ to 32.0 mm^2^ in the MRM, and from 186.5 mm^2^ to 160.4 mm^2^ in the TRM (paired *t*-test IRM: *p* = 3.27 × 10^−4^, MRM: *p* = 1.47 × 10^−3^, TRMA: *p* = 1.77 × 10^−5^). The CSA of the SRM decreased non-significantly from 41.1 mm^2^ to 37.2 mm^2^ before and after treatment (paired *t*-test: *p* = 0.069). The CSA of the LRM decreased non-significantly from 52.8 mm^2^ to 48.0 mm^2^ before and after treatment (paired *t*-test: *p* = 0.051). In the 9 g group, before and after the treatment, the CSAs of the EOMs significantly decreased from 52.4 to 32.6 mm^2^ in the IRM, from 46.6 mm^2^ to 40.0 mm^2^ in the LRM, and from 160.5 mm^2^ to 128.5 mm^2^ in the TRM (paired *t*-test IRM: *p* = 1.05 × 10^−7^, LRM: *p* = 2.10 × 10^−4^, TRMA: *p* = 1.38 × 10^−6^). The CSA of the SRM decreased non-significantly from 32.0 mm^2^ to 28.6 mm^2^ before and after treatment (paired *t*-test: *p* = 0.24). The CSA of the MRM decreased non-significantly from 29.5 mm^2^ to 27.2 mm^2^ before and after treatment (paired *t*-test: *p* = 0.15) (Figure 3).

**Figure 4:**
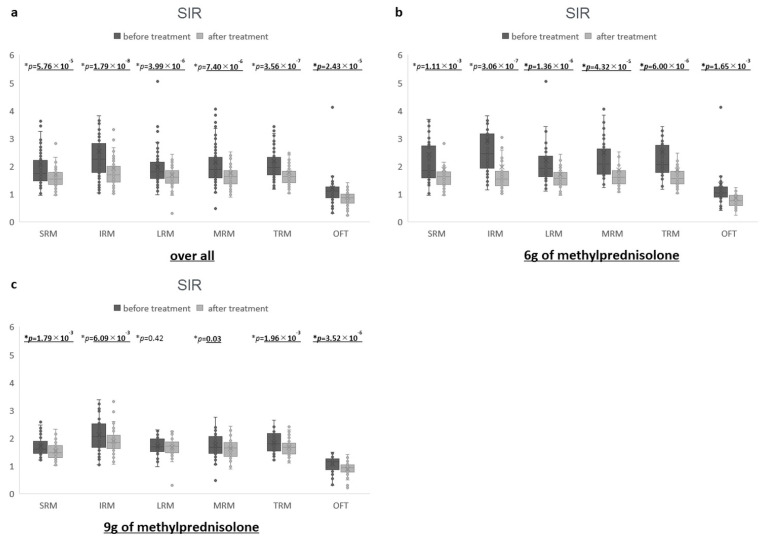
Comparison of SIR parameters before and after treatment: (**a**) overall (SRM, IRM, LRM, MRM, TRM: average, OFT), (**b**) 6 g of methylprednisolone group (SRM, IRM, LRM, MRM, TRM: average, OFT), and (**c**) 9 g of methylprednisolone group (SRM, IRM, LRM, MRM, TRM: average, OFT).

Before and after the treatment, the SIRs of the EOMs significantly decreased from 2.1 ± 1.6 to 1.7 ± 1.6 in the SRM, from 2.5 ± 1.9 to 1.9 ± 1.9 in the IRM, from 2.0 ± 1.0 to 1.7 ± 0.9 in the LRM, from 2.1 ± 1.3 to 1.8 ± 1.3 in the MRM, from 2.2 ± 1.4 to 1.8 ± 1.4 in the TRM, and from 1.2 ± 1.3 to 0.9 ± 0.7 in the OFT (paired *t*-test SRM: *p* = 5.76 × 10^−5^, IRM: *p* = 1.79 × 10^−8^, LRM: *p* = 3.99 × 10^−6^, MRM: *p* = 7.40 × 10^−6^, TRM: *p* = 3.56 × 10^−7^, OFT: *p* = 2.43 × 10^−5^) (Figure 4). In the 6 g group, before and after the treatment, the SIRs of the EOMs significantly decreased from 2.4 ± 2.1 to 1.9 ± 2.2 in the SRM, from 2.9 ± 2.0 to 2.0 ± 2.6 in the IRM, from 2.2 ± 1.3 to 1.7 ± 1.2 in the LRM, from 2.5 ± 1.7 to 1.9 ± 1.8 in the MRM, from 2.5 ± 1.9 to 1.9 ± 1.9 in the TRM, and from 1.3 ± 1.7 to 0.9 ± 0.9 in the OFT (paired *t*-test SRM: *p* = 1.11 × 10^−3^, IRM: *p* = 3.06 × 10^−7^, LRM: *p* = 1.36 × 10^−6^, MRM: *p* = 4.32 × 10^−5^, TRM: *p* =6.00 × 10^−6^, OFT: *p* = 1.65 × 10^−3^). In the 9 g group, before and after the treatment, the SIRs of the EOMs significantly decreased from 1.7 ± 0.4 to 1.5 ± 0.3 in the SRM, from 2.1 ± 0.6 to 1.9 ± 0.4 in the IRM, from 1.8 ± 0.4 to 1.6 ± 0.3 in the MRM, from 1.8 ± 0.4 to 1.7 ± 0.3 in the TRM, and from 1.1 ± 0.3 to 0.9 ± 0.3 in the OFT (paired *t*-test SRM: *p* = 1.79 × 10^−3^, IRM: *p* = 6.09 × 10^−3^, MRM: *p* = 0.03, TRM: *p* = 1.96 × 10^−3^, OFT: *p* = 3.52 × 10^−6^). The SIR of the LRM decreased non-significantly from 1.7 ± 0.3 to 1.7 ± 0.3 before and after treatment (paired *t*-test: *p* = 0.42). The SOV was identified on images in 96 of 99 eyes. The diameter of the SOV before treatment was 1.4 ± 0.4 mm. The diameter of the SOV after treatment was 1.2 ± 0.4 mm, a significant decrease from before treatment (*p* = 0.05 × 10^−5^). The dilation of the SOV was observed before treatment (defined as >2 mm) in 9 eyes (9.4%), and all eyes improved after treatment. The dilation of the SOV was observed after treatment in two eyes (2.1%), and one eye improved from before treatment while the other eye worsened. The one eye that worsened showed improvement in the CSA and SIR for all EOMs and also showed a decrease in IOP.

The degree of proptosis was identified on images in 97 of 99 eyes. Two eyes could not be analyzed because there were no axial images. The degree of proptosis after treatment was 21.5 ± 3.1 mm and the degree of exophthalmos before treatment was 22.1 ± 3.0 mm. There was a significant improvement in the degree of proptosis after treatment compared to before treatment (*p* = 0.03 × 10^−3^).

**Figure 5:**
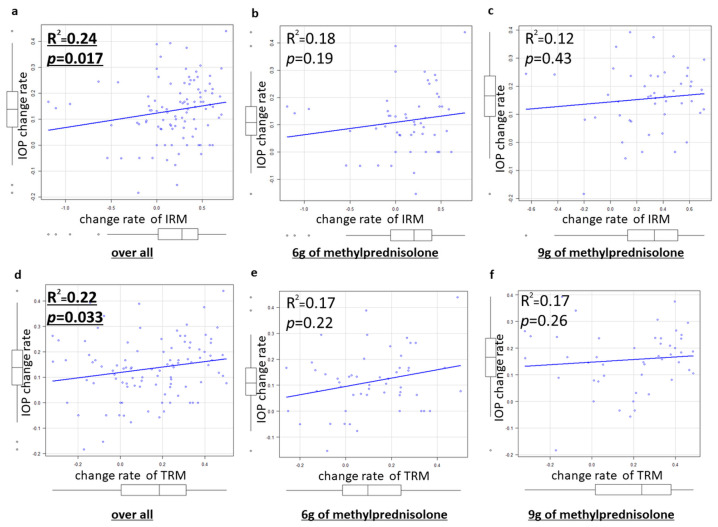
Spearman’s rank correlation coefficient (the IOP change rate versus the CSA change rate of the IRM or TRM): (**a**) overall (versus IRM), (**b**) 6 g of methylprednisolone group (versus IRM), (**c**) 9 g of methylprednisolone group (versus IRM), (**d**) overall (versus TRM), (**e**) 6 g of methylprednisolone group (versus TRM), and (**f**) 9 g of methylprednisolone group (versus TRM).

Results are shown for the correlation between the IOP change rate and the CSA change rate in of the EOMs (Spearman’s correlation coefficient). There was a weak but significant positive correlation between the CSA change rate of the IRM or TRM and the IOP change rate (IRM: R^2^ = 0.24 *p* = 0.017, TRM: R^2^ = 0.22 *p* = 0.033) (Figure 5). There was no correlation between the CSA change rate of the MRM, the SRM, or the LRM and the IOP change rate (MRM: *p* = 0.44, SRM: *p* = 0.84, LRM: *p* = 0.10). There was no significant correlation between the SIR change rate of the total EOM area and the IOP change rate. In the 6 g group, there was no significant correlation between the SIR change rates of the EOMs or the CSA change rates of the EOMs and the IOP change rate. In the 9 g group, there was no significant correlation between the SIR change rates of the EOMs or the CSA change rates of the EOMs and the IOP change rate. However, in both groups, the CSA change rates of the IRMs and the IOP change rate tended to have a positive correlation (6 g group: R^2^ = 0.18 *p* = 0.19, 9 g group: R^2^ = 0.12 *p* = 0.43). There was no significant correlation between the rate of change in the SOV diameter before and after treatment and the IOP change rate (R^2^ = −0.8 × 10^−3^ *p* = 0.99) or the CSA change rates of the total EOMs (R^2^ = −0.05 *p* = 0.62). There was no significant correlation between the rate of change in the degree of proptosis and the IOP change rate (R^2^ = 0.01, *p* = 0.53) or the CSA change rate of the total EOMs (R^2^ = 0.06, *p* = 0.90).

## 4. Discussion

This study revealed that corticosteroid therapy in conjunction with orbital radiotherapy significantly lowers the IOP in TED patients. Both the 6 g group and the 9 g group showed a significant decrease in IOP. Although there had been a study that showed that there was a significant reduction in the IOP in an ocular hypertension (OHT) group (44 eyes, IOP ≥ 20 mmHg), the study also showed that there was no significant reduction in the IOP in a non-OHT group (54 eyes, IOP < 20 mmHg) in moderate-to-severe TED patients [9]. In our current detailed study, we found that there was a significant decrease in the IOP observed in both the high IOP group and the normal IOP group. This may have been because the number of cases in our study was larger in conjunction with the combined use of orbital radiotherapy that may have further suppressed the orbital inflammation and lowered the IOP. Shams et al. have also reported that combining corticosteroid therapy with orbital radiotherapy reduces the risk of compressive optic neuropathy [14]. There was no correlation between before-treatment SE and before-treatment IOP or the IOP change rate.

In addition, the SIRs of the OFT and EOMs were significantly decreased before and after treatment. The CSAs of the EOMs were significantly decreased before and after treatment. This objectively demonstrated that combining corticosteroid therapy with orbital radiotherapy reduces EOM swelling and reduces orbital inflammation. Although we did not analyze clinical measures such as the clinical activity score (CAS) in this study, it had previously been reported that there was a significant correlation between the SIR values obtained by the MRI and CAS [15,16]. In the 6 g group and 9 g group, no significant decrease was observed in some EOMs. However, all parameters decreased after treatment compared to before treatment. Therefore, we believe that when the number of cases in each group is larger, the parameters that were not significant in this study will show a significant decrease.

Furthermore, the IRM tended to have the largest CSA and the highest SIR among the EOMs before treatment. A study using CT images had also shown that the IRM was the most swollen in TED patients, and this result was consistent with our present findings [17]. We observed that there was no significant correlation between the IOP changes and SIR changes in the OFT and EOMs.

We also found that there was a weak but significant correlation between the CSA change rate of the IRM and the IOP change rate. This suggests that the IOP decreases by reducing the swelling of the EOMs. Because the intraorbital volume is maintained constant by the seven bones, the swelling of the EOMs compresses the eyeball. As a result, the scleral veins are compressed, increasing scleral venous pressure and creating resistance to aqueous humor outflow.

This is thought to result in an increase in the IOP. Orbital decompression supports this hypothesis. A report of a CT scan performed after orbital decompression showed that the volume of the MRM was expanded [18]. Despite this, there were many reports that the IOP was significantly lowered after 2 months after performing orbital decompression [19]. Moreover, the IOP was also significantly lowered by orbital decompression at 3 months after surgery [20]. Additionally, Ye et al. reported that the swelling of the EOMs resulted in a higher IOP than that seen with the swelling of the OFT [17]. Although there was no correlation between OFT inflammation and the IOP in this study, there was a correlation between changes in the CSAs of EOMs and IOP, which increases the possibility that the above hypothesis (physical pressure causes an increase in IOP) is correct.

In addition, as a mechanism related to lowering the IOP, it has also been suggested that there is a “set value” for the episcleral venous pressure controlled by the brainstem, as reported by Jeong et al [18,21,22]. By relieving the mechanical compression of the episcleral vein, this increases blood flow to the episcleral vein and thus lowers the “set value” of the superior scleral vein pressure, thereby resulting in a decrease in the IOP [23]. This mechanism has been considered for orbital decompression surgery, and it is thought that a similar phenomenon occurs in the lowering of the IOP due to the improvement of the EOM swelling.

Another possible mechanism of increased IOP is the restriction of aqueous humor outflow to Schlemm’s canal. A previous study reported that the cross-sectional area of Schlemm’s canal is smaller and the IOP is higher in TED patients than in a healthy control group [24]. Increased IOP in TED may be due to a combination of factors including mechanical compression and outflow restriction to Schlemm’s canal.

There are some hypotheses as to why only the IRM is significantly correlated with IOP. One is that the IRM tends to swell the most, making it easier to obtain a significant correlation. The second hypothesis is that the inflow to the episcleral vein is most concentrated inferiorly so the inferior episcleral veins are most affected by pressure from the IRM swelling. In fact, it has been reported that the collector channel is mostly distributed inferiorly [25], so the greatest amount of aqueous humor may flow inferiorly into the episcleral vein.

Furthermore, one of the results of our current study was that our findings suggested that the IOP in TED patients may be reduced by anti-inflammatory therapy alone. Thus, there may be no benefit to using glaucoma eye drops without anti-inflammatory treatment in patients with ocular hypertension due to TED. Glaucoma eye drops are considered economically to constitute a social cost. In addition, there are adverse events that can occur on the patient’s ocular surface or as a systemic side effect [26,27,28]. Therefore, we believe that the fact that the IOP reduces even without the use of glaucoma eye drops is a significant finding described in our current report.

There have been reports that SOV blood flow velocity (SOV-BFV) is negatively correlated with IOP and that IOP and SOV-BFV increases after orbital decompression [23,29]. So we investigated the correlation between IOP and the diameter of the SOV but found no significant correlation in this study. This suggests that there are large individual differences in the visualization of the SOV in coronal sections using the STIR method and that it is extremely small and may have a large error.

There were several limitations to our current study. First, there was an evaluation of both eyes in a single subject. Ideally, one eye per person should be included. However, the left and right orbital tissues are independent. In TED, the orbital and muscular changes are often asymmetrical. Therefore, we decided to evaluate both eyes based on the findings of one examiner.

Second, the evaluation of the EOM and OFT inflammation by MRI was performed using only one slice. However, the muscles and fat tissue in the orbit naturally have a three-dimensional structure, and thus, there is a possibility that there were areas with strong inflammation or swelling other than in the slices that we measured during our observations. However, the evaluation of all of the slices would have taken a huge amount of time and was not realistic. As a result, we chose the currently used definition. Therefore, inflammation and EOM swelling may have been underestimated or overestimated in our current study. Additionally, since the axial length of the eyeball was not measured, the cross-section of the muscles evaluated may have been slightly different due to the difference in the axial length. Although we confirmed that the SE had no correlation with IOP, the IOP change rate, and the change rates of EOMs, it is possible that differences due to the axial length may not have been ascertained. The third limitation involved the method used to measure the IOP. The time and day of the week for measuring IOP have yet to be standardized. Currently, measurements of any diurnal fluctuation and corneal thickness are not taken into data sets for the accuracy of IOP measurement. Thus, it may have been possible to investigate whether there were any changes in corneal thickness before and after treatment in this study. A previous study reported that although there was no significant difference in corneal thickness between TED patients and a healthy control group, there was a significant difference in corneal hysteresis [30]. Measuring IOP with a Goldman tonometer will improve accuracy. Additionally, we measured IOP only in the primary position of gaze. There have been reports that IOP increases with an upward gaze [31]. The eyeball was deviated downward due to upward rotation disorder, and although it appeared to be in the primary position of gaze when measuring intraocular pressure, in reality, upward rotation was required, which could have increased the IOP before treatment. Orbital radiotherapy has the effect of improving eye movement in TED [32,33], so it is possible that the IOP decreased due to the improvement of the downward deviation of the eye after treatment. The fourth limitation was the treatment. In our study, all patients with active TED were treated with corticosteroids and orbital radiotherapy. Thus, we could not compare the two control groups of patients treated with corticosteroids only and orbital radiotherapy only. Therefore, we cannot conclude whether the factors responsible for the improvement in IOP were due to corticosteroids, orbital radiotherapy, or both.

In recent years, teprotumumab has been approved in Japan for the treatment of active TED. Teprotumumab is a groundbreaking drug that improves exophthalmos and diplopia by inhibiting insulin-like growth factor 1 receptor (IGF-1R). IGF-1R is overexpressed in orbital fibroblasts [34], so it may reduce orbital pressure more than methylprednisolone and contribute to lowering IOP. However, teprotumumab can also cause side effects such as muscle cramps, ototoxicity, alopecia, fatigue, and hyperglycemia. In particular, ototoxicity often does not improve, and the drug price is high [35]. In the future, methylprednisolone may be replaced by teprotumumab in cases of severe TED with ocular hypertension, exophthalmos, and diplopia. But we believe that it is necessary to explain the advantages and disadvantages of both methyOKlprednisolone and teprotumumab to patients and choose carefully when administering them.

## 5. Conclusions

In patients with TED, there was a decrease in IOP with anti-inflammatory treatment alone, and there were very few cases where glaucoma eye drops were additionally used. The most significant parameter that was correlated with the decrease in the IOP was the volume change rate of the IRM. Therefore, the assessment of EOM swelling before treatment may enable the prediction of IOP changes after treatment.

## Figures and Tables

**Table 1:** Patient background (before treatment).

	Overall	6 g of Methylprednisolone	9 g of Methylprednisolone
**patients**	51	27	24
**sex**	Male: 11; female: 40	Male: 7; female: 20	Male: 4; female: 20
**eyes**	99 (right: 49; left: 50)	52 (right: 25; left: 27)	47 (right: 24; left: 23)
**Average age**	54.5 ± 12.9 (18–83)	53.8 ± 12.5 (29–77)	54.3 ± 12.8 (18–83)
**IOP (mmHg)**	18.0 ± 3.4 (9.5–30.3)	17.9 ± 3.7 (10–30.3)	18.2 ± 2.9 (9.5–25)
**Spherical equivalent (D)**	−2.4 ± 2.8 (−10.9 ± 2.3)	−2.2 ± 2.9 (−10.9 ± 2.3)	−2.6 ± 2.7 (−10.1 ± 2.0)
**Number of patients** **per refraction(below)**			
**Emmetropia, hyperopia** **(≥0D)**	23 (23.2%)	15 (28.9%)	8 (17.0%)
**Mild myopia** **(>−3.0D, <0D)**	39 (39.4%)	18 (34.6%)	21 (44.7%)
**Moderate myopia** **(>−6.0D, ≤−3.0D)**	27 (27.3%)	13 (25.0%)	14 (29.8%)
**Severe myopia** **(≤−6.0D)**	10 (10.1%)	6 (11.5%)	4 (8.5%)
**Thyroid function (FT4)**			
**Hypothyroidism** **(FT4 ≤ 0.9 ng/dL)**	18 (18.9%)	16 (16.8%)	2 (2.1%)
**Euthyroid** **(FT4 = 0.9–1.7 ng/dL)**	63 (66.3%)	30 (31.6%)	33 (34.7%)
**Hyperthyroidism** **(FT4 ≥ 1.7 ng/dL)**	14 (14.7%)	6 (6.3%)	8 (8.4%)

## Data Availability

All data generated or analyzed during this study have been included in this published article.
